# Supplementary material for: Insight into the PmrB structures of colistin-resistant Gram-negative bacteria through the multi-template ligand-guided homology modeling and in silico mutagenesis
Source: PeerJ. 2025 Sep 3;13:e19945. doi: 10.7717/peerj.19945 (PMC12422264; doi:10.7717/peerj.19945)
Supplement: Supplemental Information 1 — (A) Models ranked by binding energy from ligand-guided homology modeling. (B) Models ranked by DOPE score from conventional multi-template homology modeling. The aligned structures are highlighted by their RMSD values, with dark blue indicating low RMSD and red indicating high RMSD compared to the top-ranked model from each approach. [file peerj-13-19945-s001.docx]

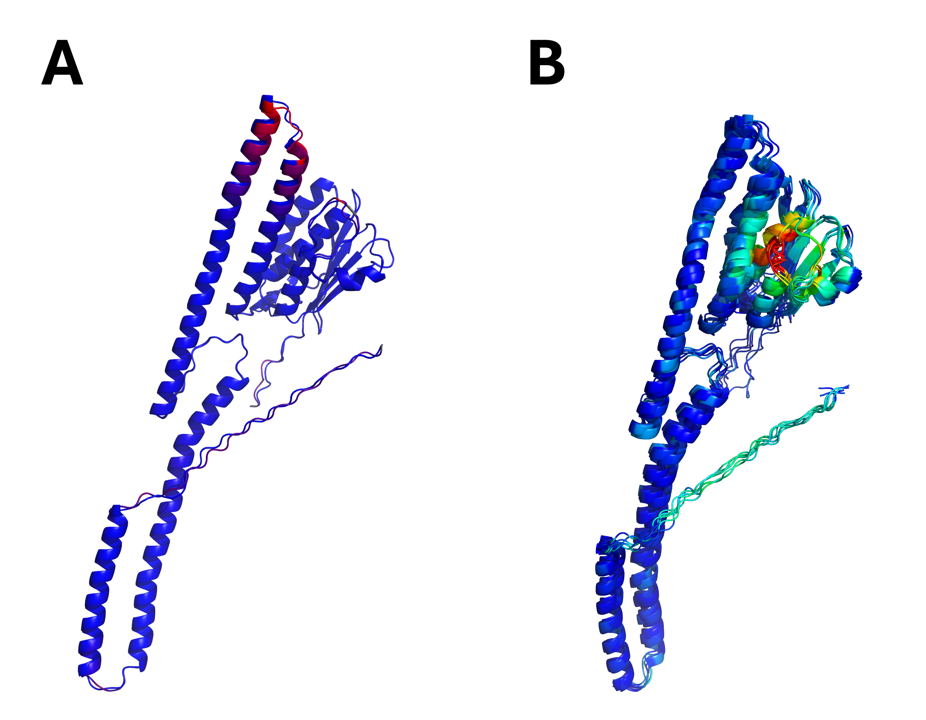


**Figure S1.** **A comparison of the top-ranked PmrB models of *Klebsiella pneumoniae* in monomeric form.** (A) Models ranked by binding energy from ligand-guided homology modeling. (B) Models ranked by DOPE score from conventional multi-template homology modeling. The aligned structures are highlighted by their RMSD values, with dark blue indicating low RMSD and red indicating high RMSD compared to the top-ranked model from each approach.
