# Supplementary material for: Insight into the PmrB structures of colistin-resistant Gram-negative bacteria through the multi-template ligand-guided homology modeling and in silico mutagenesis
Source: PeerJ. 2025 Sep 3;13:e19945. doi: 10.7717/peerj.19945 (PMC12422264; doi:10.7717/peerj.19945)
Supplement: Supplemental Information 3 — (A) Root Mean Square Deviation (RMSD) of the PmrB protein structures for A. baumannii, E. coli, K. pneumoniae, and P. aeruginosa. (B) RMSD of the ATP ligand within the PmrB ATP-binding site. (C) Root Mean Square Fluctuation (RMSF) of PmrB residues, indicating the flexibility of different regions of the protein. (D) Radius of Gyration (Rg) of PmrB, reflecting the compactness of the protein structure over time. (E) Solvent Accessible Surface Area (SASA) of PmrB, showing the protein’s exposed surface area to the solvent. (F) Number of hydrogen bonds formed between PmrB and the ATP ligand during the simulation. [file peerj-13-19945-s003.docx]

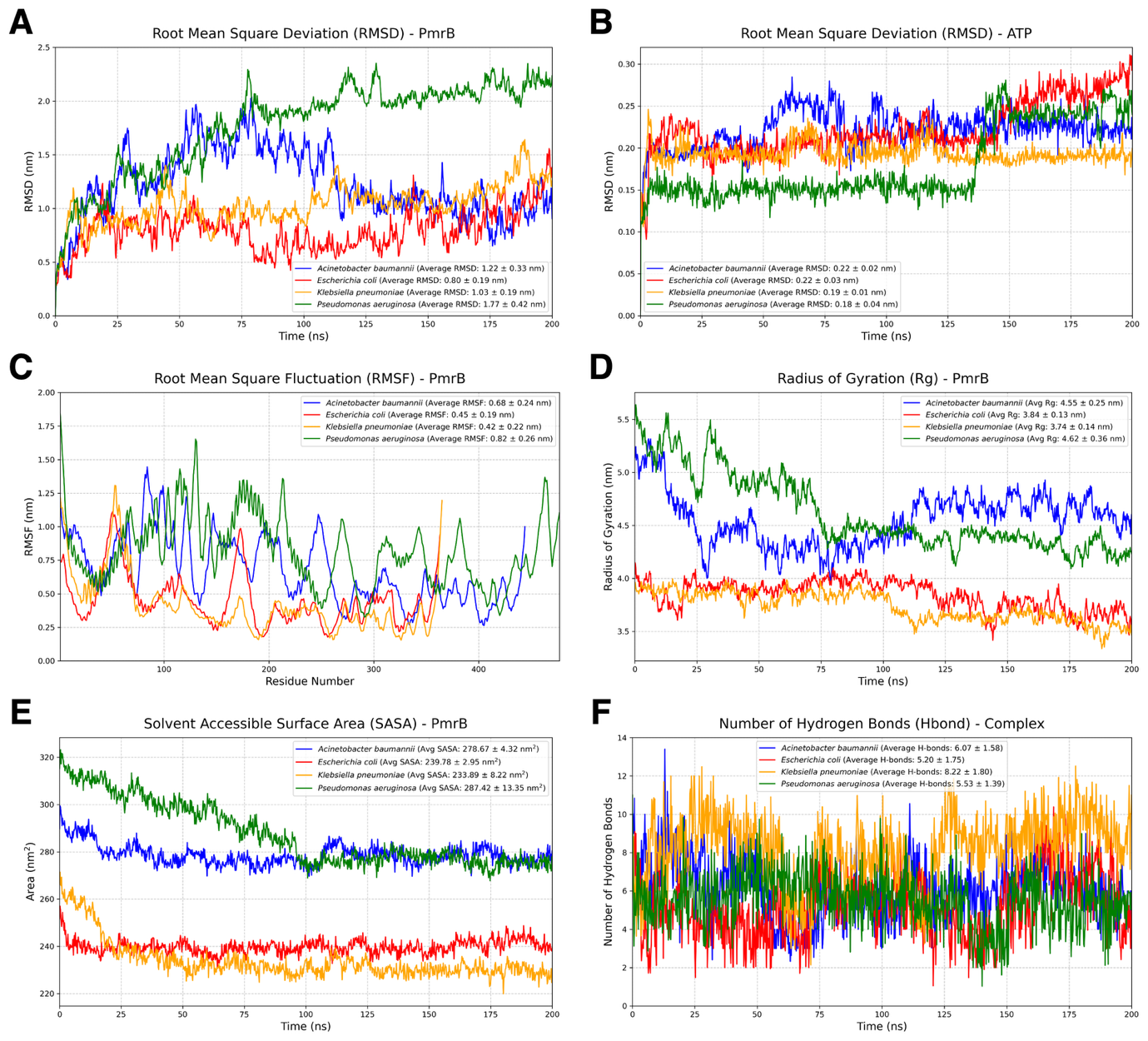


**Figure S3. Analysis of the second molecular dynamics (MD) trajectory of PmrB homology models.** (A) Root Mean Square Deviation (RMSD) of the PmrB protein structures for *A. baumannii*, *E. coli*, *K. pneumoniae*, and *P. aeruginosa*. (B) RMSD of the ATP ligand within the PmrB ATP-binding site. (C) Root Mean Square Fluctuation (RMSF) of PmrB residues, indicating the flexibility of different regions of the protein. (D) Radius of Gyration (Rg) of PmrB, reflecting the compactness of the protein structure over time. (E) Solvent Accessible Surface Area (SASA) of PmrB, showing the protein's exposed surface area to the solvent. (F) Number of hydrogen bonds formed between PmrB and the ATP ligand during the simulation.
