# Supplementary material for: Insight into the PmrB structures of colistin-resistant Gram-negative bacteria through the multi-template ligand-guided homology modeling and in silico mutagenesis
Source: PeerJ. 2025 Sep 3;13:e19945. doi: 10.7717/peerj.19945 (PMC12422264; doi:10.7717/peerj.19945)
Supplement: Supplemental Information 5 — The left panel (A–D) represents energetic components contributing to the total binding free energy (ΔG), which were calculated using the MMGBSA method for A. baumannii (A), E. coli (B), K. pneumoniae (C), and P. aeruginosa (D). Energy components include van der Waals (VDWAALS), electrostatic (EEL), polar solvation (EGB), and nonpolar solvation (ESURF) energies. The right panel (E-H) displays per-residue decomposition analysis of the PmrB-ATP binding free energy for A. baumannii (E), E. coli (F), K. pneumoniae (G), and P. aeruginosa (H) highlighting individual residues’ energetic contribution to the PmrB-ATP interaction, where the residues with negative values contribute favorably to the binding. [file peerj-13-19945-s005.docx]

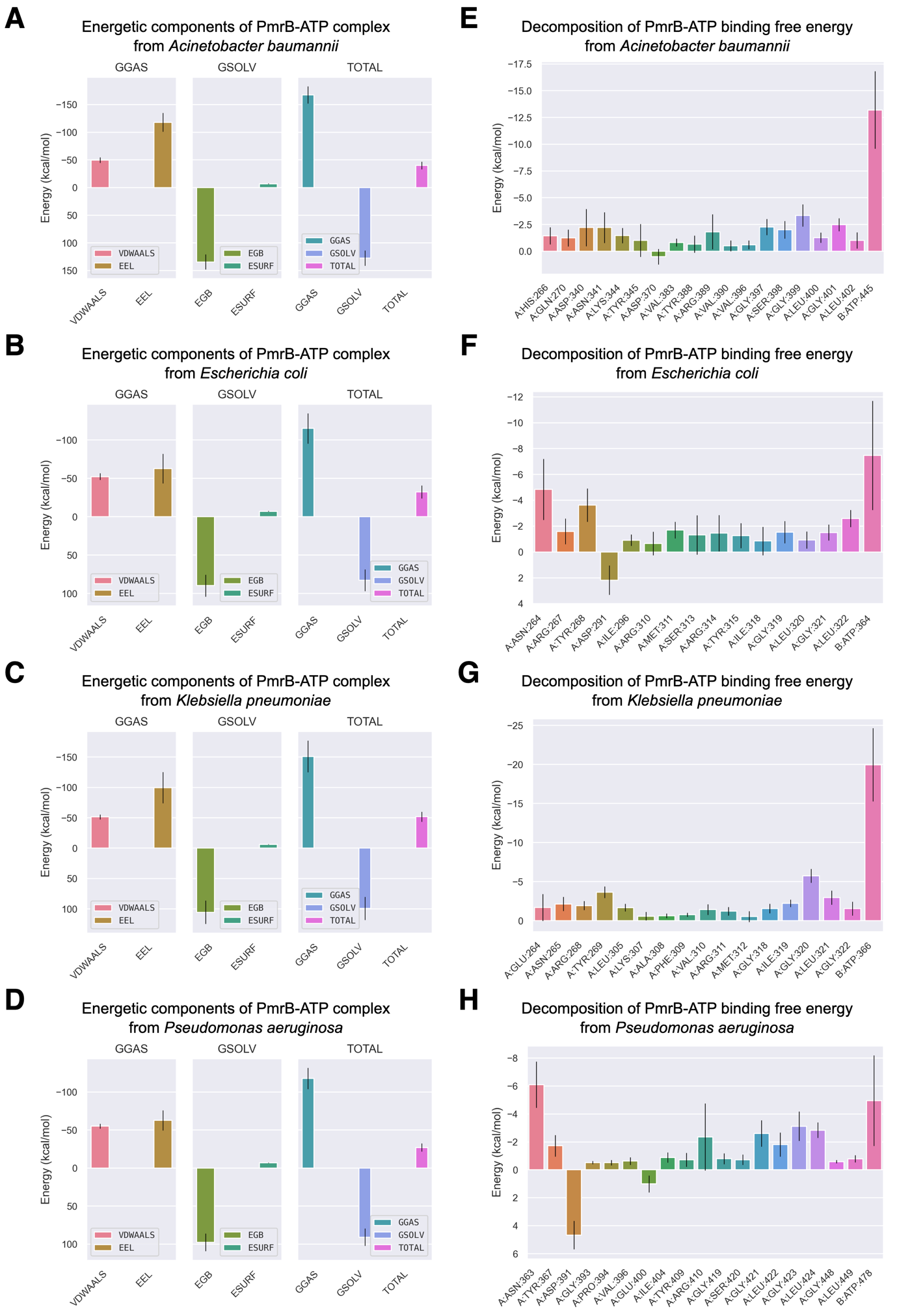


**Figure S5. Analysis of binding free energy components and per-residue decomposition from the second molecular dynamics (MD) trajectory of PmrB-ATP interaction.** The left panel (A-D) represents energetic components contributing to the total binding free energy (ΔG), which were calculated using the MMGBSA method for *A. baumannii* (A), *E. coli* (B), *K. pneumoniae* (C), and *P. aeruginosa* (D). Energy components include van der Waals (VDWAALS), electrostatic (EEL), polar solvation (EGB), and nonpolar solvation (ESURF) energies. The right panel (E-H) displays per-residue decomposition analysis of the PmrB-ATP binding free energy for *A. baumannii* (E), *E. coli* (F), *K. pneumoniae* (G), and *P. aeruginosa* (H) highlighting individual residues' energetic contribution to the PmrB-ATP interaction, where the residues with negative values contribute favorably to the binding.
