# Supplementary material for: Insight into the PmrB structures of colistin-resistant Gram-negative bacteria through the multi-template ligand-guided homology modeling and in silico mutagenesis
Source: PeerJ. 2025 Sep 3;13:e19945. doi: 10.7717/peerj.19945 (PMC12422264; doi:10.7717/peerj.19945)
Supplement: Supplemental Information 6 — The equilibrated structure corresponds to the frame with the lowest ΔG after 50 ns equilibration. (A) A. baumannii. (B) E. coli. (C) K. pneumoniae. (D) P. aeruginosa. Left panel: initial structure (green); Middle panel: lowest ΔG structure (white); Right panel: superimposition. This figure demonstrates the stability of the PmrB models and highlights conformational changes upon equilibration, particularly in loop regions and the ATP-binding site. The close agreement between structures supports the reliability of the models for further investigations. [file peerj-13-19945-s006.docx]

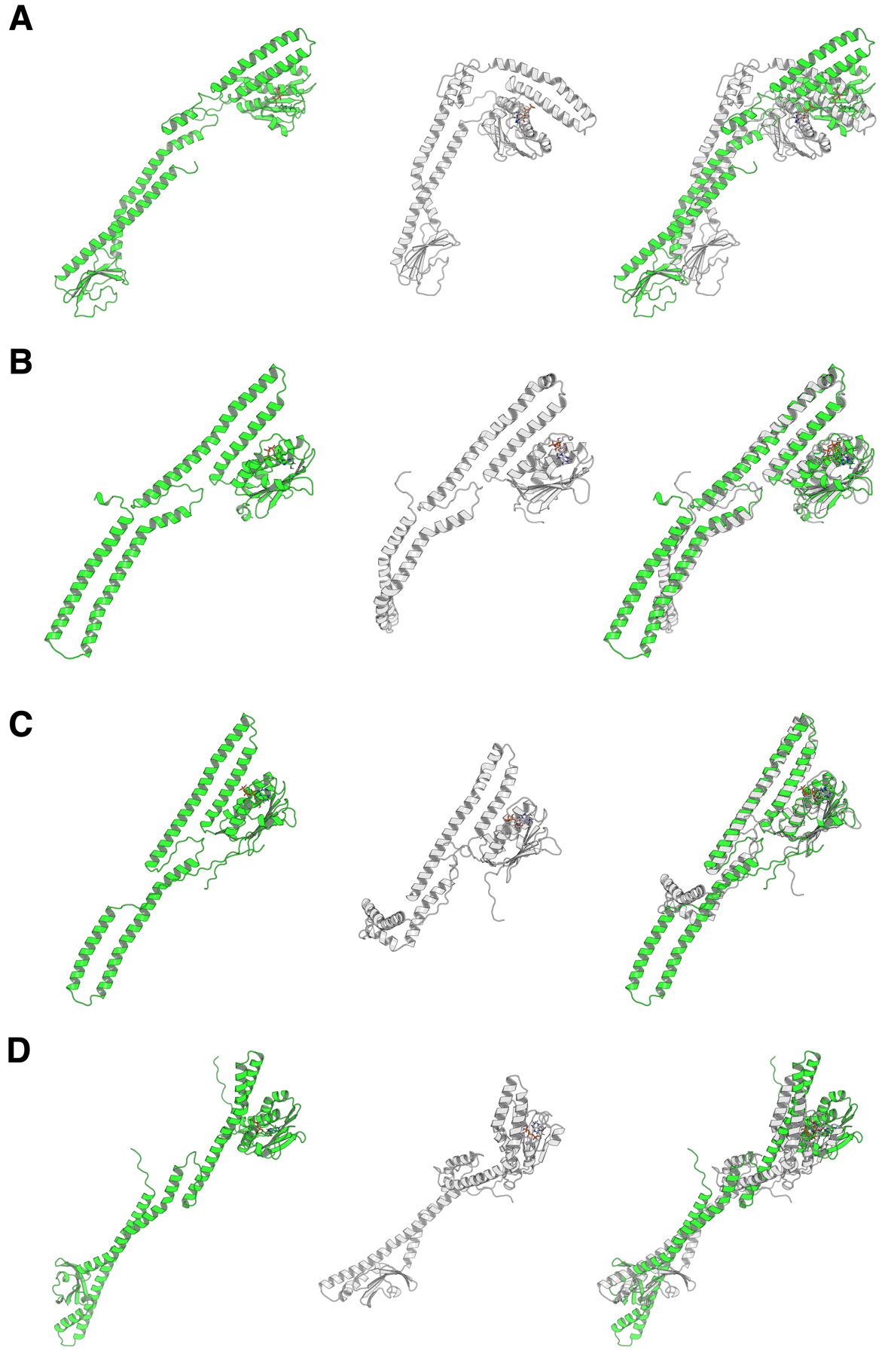


**Figure S6. Comparison of initial and equilibrated PmrB homology models from MD simulations.** The equilibrated structure corresponds to the frame with the lowest ΔG after 50 ns equilibration. (A) *A. baumannii*. (B) *E. coli*. (C) *K. pneumoniae*. (D) *P. aeruginosa*. Left panel: initial structure (green); Middle panel: lowest ΔG structure (white); Right panel: superimposition. This figure demonstrates the stability of the PmrB models and highlights conformational changes upon equilibration, particularly in loop regions and the ATP-binding site. The close agreement between structures supports the reliability of the models for further investigations.
