# Supplementary material for: Insight into the PmrB structures of colistin-resistant Gram-negative bacteria through the multi-template ligand-guided homology modeling and in silico mutagenesis
Source: PeerJ. 2025 Sep 3;13:e19945. doi: 10.7717/peerj.19945 (PMC12422264; doi:10.7717/peerj.19945)
Supplement: Supplemental Information 7 [file peerj-13-19945-s007.docx]

**Table S1.** **Amino acid sequences of selected bacterial PmrB for constructing ligand-guided homology models.**

| **Organisms** | **Accession Number** | **Amino Acid Sequence** |  |
| --- | --- | --- | --- |
|  |  |  |  |
| *Klebsiella pneumoniae* | WP_004886129.1 | MALFATENWTMRHRLLLTIGAILVVCQLISVFWLWHESKEQIQLLVASAIEGHNNQKHVEHEVREAVASLLVPSLLIVGLALYISMLAVRKITRPLSRLQSELESRTPDNLTPIVLSESVPEVTAVTTALNQLVSRLNLTLDRERLFTADVAHELRTPLAGLRLHLELLAKVHGMGVDPLIQRLDQMTTSISQLLQLARVGQSFSAGSYQQVLLLDDVVKPLQDELETMLAERQQRLLLTDVESEAVVSGDATLIRVILRNLVENAHRYSPQGSTIRVSVKAGLMPVMAVEDEGPGIDEAKSGELSKAFVRMDSRYGGIGLGLSIVTRIAQLHDAQFFLHNRQPGPGVRAWVLFPRRGGQNVSTH |  |
| *Escherichia coli* | QIR30685.1 | MRFLRRPISLRQRLILTIGAILLVFELISVFWLWHESTEQIQLFEQALRDNRNNDRHIMREIREAVASLIVPGVFMVSLTLFICYQAVRRITRPLAELQKELEARTADNLTPIAIHSATLEIDAVVSALNDLVSRLTSTLDNERLFTADVAHELRTPLAGVRLHLELLAKTHHIDVAPLVARLDQMMESVSQLLQLARAGQSFSSGNYQHVKLLEDVILPSYDELSTMLDQRQQTLLLPESAADITVQGDATLLRMLLRNLVENAHRYSPQGSNIMIKLQEDGGAVMAVEDEGPGIDESKCGELSKAFVRMDSRYGGIGLGLSIVSRITQLHHGQFFLQNRQETSGTRAWIRLKKDQYVANQI |  |
| *Pseudomonas aeruginosa* | AAG08163.1 | MSRAAVPSVRRRLLVNLLVGFVLCWLSVAALTYHLSLKQVNRLFDDDMVDFGEAALRLLDLATEDQAGEDGSITEIIERSREAIQGLPLLRRESALGYALWRDGQPLLSSLNLPPEITAQGPGFSTVEAQGTHWRVLQLNIDGFQIWISENLIYRQHTMNLLLFYSLFPLLLALPLLGGLVWFGVARGLAPLREVQAEVQQRSARHLQPIAVEAVPLEIRGLIDELNLLLERLRTALEAERRLTSDAAHEIRTPLASLRTHAQVALRSEDPKAHARGLLQVSRSVERISTLMEQILLLARLDGDALLEQFHPVNLATLAEDVLSELARQAIDKDIELSLHQETVYVMGIDLWLKAMVGNLVGNALRYTPAGGQVEIRVENRAQHAVLRVRDNGPGVALEEQQAIFTRFYRSPATSSGEGSGLGLPIVKRIVELHFGSIGLGKGLEGKGLEVQVFLPKTQPDATRPPARGPDSGRSHI |  |
|  |  |  |  |
|  |  |  |  |
| *Acinetobacter baumannii* | ADK46865.1 | MHYSLKKRLIWGTSIFSVILGCILIFSAYKVALQEVDEILDTQMKYLAERTAEHPLKTVSSKFDFHKTYHEEDLFIDIWAYKDQAHLSHHLHLLVPPVEQAGFYSHKTAQGIVRTYVLPLKDYQIQVSQQERVREAFAWELAGSMFIPYLIILPFAIFALAAIIRRGLKPIDDFKNELKERDSEELTPIEVHDYPQELLPTIDEMNRLFERISKAQNEQKQFIADAVHELRTPVTALNLQTKILLSQFPEHESLQNLSKGLARIQHLVTQLLALAKQDVTLSMVEPTGYFQLNDVALNCVEQLVNLAMQKEIDLGFVRNEPIEMHSIEPTVHSIIFNLIDNAIKYTPHQGVINISVYTDPDHYACIQIEDSGAGIDPENYDKVLKRFYRVHHHLEVGSGLGLSIVDRATQRLGGTLTLDKSLELGGLSVLVKLPKVLHLNETRA |  |
|  |  |  |  |
|  |  |  |  |
|  |  |  |  |
|  |  |  |  |
