# Supplementary material for: Insight into the PmrB structures of colistin-resistant Gram-negative bacteria through the multi-template ligand-guided homology modeling and in silico mutagenesis
Source: PeerJ. 2025 Sep 3;13:e19945. doi: 10.7717/peerj.19945 (PMC12422264; doi:10.7717/peerj.19945)
Supplement: Supplemental Information 8 [file peerj-13-19945-s008.docx]

**Table S2.** **The amino acid position of PmrB substructural domains.**

| **Substructure** | **Amino acid position (total number of amino acid)** | | | | | | | |
| --- | --- | --- | --- | --- | --- | --- | --- | --- |
|  | ***Acinetobacter baumannii*** | | ***Escherichia coli*** | | ***Klebsiella pneumoniae*** | | ***Pseudomonas aeruginosa*** | |
| Signal peptide | 1-6 | (6) | 1-11 | (12) | 1-14 | (14) | 1-12 | (12) |
| Transmembrane 1 | 9-29 | (21) | 12-34 | (23) | 15-35 | (21) | 13-33 | (21) |
| Extracellular sensor | 30-145 | (116) | 35-64 | (30) | 36-65 | (30) | 34-160 | (127) |
| Transmembrane 2 | 146-164 | (19) | 65-85 | (21) | 66-89 | (24) | 161-185 | (25) |
| HAMP domain | 165-213 | (49) | 86-141 | (56) | 90-140 | (51) | 186-237 | (52) |
| DHp domain | 214-279 | (66) | 142-205 | (64) | 141-205 | (65) | 238-302 | (65) |
| Catalytic domain | 280-444 | (165) | 206-363 | (158) | 206-365 | (160) | 303-477 | (175) |
| ATPase lid loop | 385-400 | (16) | 305-320 | (16) | 305-320 | (16) | 405-422 | (18) |

HAMP, Histidine kinases, Adenylyl cyclases, Methyl-accepting chemotaxis proteins, and Phosphatases domain; DHp, dimerization and histidine phosphotransfer domain
