# Supplementary material for: Insight into the PmrB structures of colistin-resistant Gram-negative bacteria through the multi-template ligand-guided homology modeling and in silico mutagenesis
Source: PeerJ. 2025 Sep 3;13:e19945. doi: 10.7717/peerj.19945 (PMC12422264; doi:10.7717/peerj.19945)
Supplement: Supplemental Information 10 [file peerj-13-19945-s010.docx]

**Table S4.** **Protein-ligand interaction profile of all PmrB models in complex with ATP.**

| **Organisms** | **Moiety** | **Interaction type** | **Residue** | **Interaction site** | **Protein Substructure** |
| --- | --- | --- | --- | --- | --- |
| *Acinetobacter*  *baumannii* | Adenine | Hydrogen bond | A373 | Main chain | Catalytic domain |
|  |  | 𝜋-𝜋 stacking | Y345 | Side chain | Catalytic domain |
|  | Ribose | Hydrogen bond | Y388 | Main chain | Lid-loop |
|  |  |  | V390 | Main chain | Lid-loop |
|  | Phosphate | Salt bridge | K344 | Side chain | Catalytic domain |
|  |  |  | R389 | Side chain | Catalytic domain |
|  |  | Hydrogen bond | G397 | Main chain | Lid-loop |
|  |  |  | S398 | Main chain | Lid-loop |
|  |  |  | G399 | Main chain | Lid-loop |
|  |  |  | N341 | Side chain | Catalytic domain |
|  |  |  | T345 | Side chain | Catalytic domain |
|  |  |  | G401 | Main chain | Catalytic domain |
| *Escherichia*  *coli* | Adenine | Hydrogen bond | P294 | Main chain | Catalytic domain |
|  |  | 𝜋-𝜋 stacking | Y268 | Side chain | Catalytic domain |
|  | Ribose | Hydrogen bond | R310 | Side chain | Lid-loop |
|  |  |  | M311 | Main chain | Lid-loop |
|  |  |  | D312 | Main chain | Lid-loop |
|  |  |  | Y315 | Main chain | Lid-loop |
|  | Phosphate | Salt bridge | R310 | Side chain | Lid-loop |
|  |  | Hydrogen bond | G317 | Main chain | Lid-loop |
|  |  |  | I318 | Main chain | Lid-loop |
|  |  |  | G319 | Main chain | Lid-loop |
|  |  |  | G321 | Main chain | Catalytic domain |
|  |  |  | L322 | Main chain | Catalytic domain |
|  |  |  | N264 | Side chain | Catalytic domain |
|  |  |  | Y268 | Side chain | Catalytic domain |
| *Klebsiella*  *pneumoniae* | Adenine | Hydrogen bond | M312 | Main chain | Lid-loop |
|  |  |  | Y316 | Main chain | Lid-loop |
|  |  | 𝜋-𝜋 stacking | Y269 | Side chain | Catalytic domain |
|  | Ribose | Hydrogen bond | L305 | Main chain | Lid-loop |
|  |  |  | G320 | Main chain | Lid-loop |
|  |  |  | L321 | Main chain | Catalytic domain |
|  |  |  | G322 | Main chain | Catalytic domain |
|  |  |  | L323 | Main chain | Catalytic domain |
|  |  |  | S324 | Main chain | Catalytic domain |
|  | Phosphate | Hydrogen bond | T189 | Side chain | Dhp domain |
|  |  |  | Q193 | Side chain | Dhp domain |
|  |  |  | I319 | Main chain | Lid-loop |
|  |  |  | L321 | Main chain | Catalytic domain |
| *Pseudomonas*  *aeruginosa* | Adenine | 𝜋-𝜋 stacking | Y367 | Side chain | Catalytic domain |
|  |  | Hydrogen bond | D391 | Side chain | Catalytic domain |
|  | Ribose | Hydrogen bond | S411 | Main chain | Lid-loop |
|  |  |  | T414 | Side chain | Lid-loop |
|  | Phosphate | Hydrogen bond | S420 | Main chain | Lid-loop |
|  |  |  | G421 | Main chain | Lid-loop |
|  |  |  | L422 | Main chain | Lid-loop |
|  |  |  | G423 | Main chain | Catalytic domain |
|  |  |  | G424 | Main chain | Catalytic domain |
|  |  |  | Q294 | Side chain | Dhp domain |
