# Supplementary material for: Insight into the PmrB structures of colistin-resistant Gram-negative bacteria through the multi-template ligand-guided homology modeling and in silico mutagenesis
Source: PeerJ. 2025 Sep 3;13:e19945. doi: 10.7717/peerj.19945 (PMC12422264; doi:10.7717/peerj.19945)
Supplement: Supplemental Information 11 [file peerj-13-19945-s011.docx]

**Table S5.** **Comparison of the free energy changes in PmrB mutants of *K. pneumoniae* compared to the wild type.**

| **Mutant** | **Location** | **Total energy (kcal/mol)** | **Type of free energy contribution (kcal/mol)** | | | | | | | | | | | | |
| --- | --- | --- | --- | --- | --- | --- | --- | --- | --- | --- | --- | --- | --- | --- | --- |
|  |  |  | **Backbone HBond** | **Sidechain Hbond** | **Van der Waals** | **Electrostatics** | **Solvation polar** | **Solvation hydrophobic** | **Van der Waals clashes** | **Entropy sidechain** | **Entropy mainchain** | **Torsional clash** | **Backbone clash** | **Helix dipole** | **Energy ionisation** |
| L17Q | TM1 | -0.073 | -0.597 | -0.597 | -0.136 | 0.000 | 0.339 | 0.055 | 0.071 | 0.632 | 0.161 | 0.000 | 0.000 | 0.000 | 0.000 |
| L82R | TM2 | 0.509 | 0.020 | 0.000 | -0.051 | 0.050 | 0.079 | 0.121 | 0.009 | 0.096 | 0.122 | 0.064 | -0.119 | 0.000 | 0.000 |
| S85R | TM2 | -0.738 | -0.407 | -0.198 | -0.503 | 0.041 | 0.300 | -0.826 | -0.028 | 0.698 | 0.046 | 0.071 | 0.278 | 0.067 | 0.000 |
| T140P | HAMP | 4.177 | 1.873 | 0.589 | -0.008 | 0.000 | -0.964 | -0.574 | 4.133 | -0.276 | -0.594 | 0.000 | -2.450 | 0.000 | 0.000 |
| D150H | DHp | -0.384 | -0.737 | -0.632 | -0.385 | 0.205 | 0.544 | -0.428 | 0.001 | 0.773 | 0.274 | 0.002 | 0.259 | -0.079 | 0.077 |
| T157P | DHp | -1.113 | -0.018 | 0.000 | -0.332 | 0.000 | -0.195 | -0.735 | 0.905 | 0.269 | -1.007 | 0.000 | -0.957 | 0.000 | 0.000 |
| S205P | DHp | 1.329 | 1.354 | 0.632 | -0.107 | 0.031 | -0.621 | -0.830 | 2.560 | -0.281 | -1.417 | 0.008 | -1.187 | 0.000 | 0.000 |
| S208N | CA | -0.859 | -0.210 | -0.210 | -0.140 | 0.000 | 0.108 | -0.279 | 0.000 | 0.265 | -0.438 | 0.044 | 0.036 | 0.000 | 0.000 |
| T246A | CA | -0.934 | 0.326 | 0.289 | 0.805 | 0.000 | -1.232 | 0.855 | -1.473 | -0.551 | 0.206 | -0.160 | -0.043 | 0.000 | 0.000 |
| R256G | CA | 1.983 | 1.460 | 1.300 | 1.958 | 0.201 | -2.629 | 2.223 | -0.399 | -1.717 | 0.078 | -0.105 | -0.492 | -0.388 | 0.000 |
| K281L | CA | 0.068 | 0.694 | 0.785 | 0.072 | 0.151 | -0.691 | -0.137 | 0.010 | -0.902 | -0.040 | 0.127 | 0.107 | 0.000 | 0.000 |
| L339C | CA | 2.209 | -0.138 | -0.165 | 1.112 | 0.003 | -0.678 | 2.625 | 0.135 | -0.229 | -0.250 | -0.206 | -0.001 | 0.000 | 0.000 |
| H340I | CA | 0.859 | 0.610 | 0.632 | 0.143 | 0.009 | -0.646 | -0.295 | 0.188 | -0.558 | 0.522 | 0.334 | -0.004 | 0.000 | -0.078 |
| N341T | CA | 0.312 | 0.718 | 2.006 | 0.616 | -0.204 | -1.574 | 0.551 | -0.002 | -1.437 | -0.225 | -0.150 | 0.060 | 0.000 | 0.014 |
| R342D | CA | 2.989 | 0.451 | 0.643 | 1.062 | 1.600 | 0.153 | 0.957 | -0.112 | -1.052 | -0.531 | -0.182 | -1.288 | 0.000 | 0.000 |
| Q343S | CA | -0.632 | 0.138 | 0.111 | 0.517 | -0.083 | -0.419 | 0.560 | -0.038 | -0.667 | -0.931 | -0.006 | -0.281 | 0.000 | 0.187 |
| L344P | CA | -1.381 | 0.000 | 0.000 | 0.004 | -0.011 | -0.176 | -0.001 | -0.025 | 0.004 | -0.955 | -0.222 | -0.571 | 0.000 | 0.000 |
| P346Q | CA | 0.330 | -0.701 | -0.632 | -0.390 | 0.021 | 0.555 | -0.438 | 0.002 | 0.810 | 1.109 | -0.007 | -0.104 | 0.000 | 0.000 |
| T246A/L344P | CA | -4.300 | -0.052 | -0.075 | 0.983 | 0.001 | -1.897 | 1.226 | -0.729 | -0.556 | -0.608 | -3.717 | -0.070 | 0.000 | 0.000 |

TM1, transmembrane region 1; TM2, transmembrane region 2; HAMP, Histidine kinases, Adenylyl cyclases, Methyl-accepting chemotaxis proteins, and Phosphatases domain; DHp, dimerization and histidine phosphotransfer domain; CA, catalytic domain including ATP-binding site.
